# Role of Kallistatin Treatment in Aging and Cancer by Modulating miR-34a and miR-21 Expression

**DOI:** 10.1155/2017/5025610

**Published:** 2017-06-28

**Authors:** Julie Chao, Youming Guo, Pengfei Li, Lee Chao

**Affiliations:** Department of Biochemistry and Molecular Biology, Medical University of South Carolina, Charleston, SC, USA

## Abstract

Kallistatin is an endogenous protein that regulates differential signaling pathways and a wide spectrum of biological activities via its two structural elements: an active site and a heparin-binding domain. Kallistatin via its heparin-binding site inhibits vascular inflammation and oxidative stress by antagonizing TNF-*α*-induced NADPH oxidase activity, NF-*κ*B activation, and inflammatory gene expression in endothelial cells. Moreover, kallistatin via its active site inhibits microRNA-34a (miR-34a) synthesis and stimulates eNOS and SIRT1 expression in endothelial progenitor cells, whereas its heparin-binding site is crucial for blocking TNF-*α*-induced miR-21 expression and oxidative stress, thus reducing cellular senescence. By downregulating miR-34a and miR-21 expression, kallistatin treatment attenuates oxidative damage and aortic senescence in streptozotocin-induced diabetic mice and extends *Caenorhabditis elegans* lifespan under stress conditions. Likewise, kallistatin through the heparin-binding site inhibits TGF-*β*-induced miR-21 synthesis and oxidative stress in endothelial cells, resulting in inhibition of endothelial-mesenchymal transition, a process contributing to fibrosis and cancer. Furthermore, kallistatin's active site is essential for stimulating miR-34a and p53 expression and inhibiting the miR-21-Akt-Bcl-2 signaling pathway, thus inducing apoptosis in breast cancer cells. These findings reveal novel mechanisms of kallistatin in protection against senescence, aging, and cancer development by modulating miR-34a and miR-21 levels and inhibiting oxidative stress.

## 1. Introduction

Aging, characterized by the deterioration of human physiological functions, is the dominant risk factor for the development of cardiovascular disease and cancer [1]. Aging and cancer share common origins, as increased production of reactive oxygen species (ROS) plays a critical role in oxidative damage in aged cells and tumor progression [2, 3]. Thus, identifying new antioxidant agents to delay aging and reduce cancer development is imperative in pharmacological intervention. Moreover, microRNAs (miRNAs) negatively regulate the expression of target genes at the posttranscriptional level and play a pivotal role in cellular senescence and tumor progression [4, 5]. Among miRNAs, miR-21 has a widely described oncogenic function by targeting multiple signaling pathways in regulating cancer cell apoptosis, migration, invasion, proliferation, and angiogenesis [6, 7]. Conversely, miR-34a inhibits cancer cell survival, proliferation, invasion, and metastasis formation [8, 9]. Thus, miR-21 functions as an oncogene, while miR-34a acts as a tumor suppressor. However, both miR-34a and miR-21 promote cellular senescence and are crucial players in the regulation of aging and cancer development.

miR-21 promotes tumor progression by stimulating ROS production through downregulation of superoxide dismutase (SOD2/SOD3) [10]. Besides its association with oxidative stress, miR-21 has a potential role in fibrosis, as inhibition of miR-21 attenuates fibrosis in the heart, kidney, and lungs [11–13]. Transforming growth factor- (TGF-) *β*1, via stimulation of ROS formation, induces upregulation of miR-21, leading to organ fibrosis [14]. Moreover, miR-21 plays an important role in TGF-*β*-induced endothelial-to-mesenchymal transition (EndMT), a process that contributes to carcinoma-associated fibrosis and fibrotic disease [15]. Therefore, miR-21 exhibits a functional interplay with oxidative stress during tumorigenesis and fibrosis. Furthermore, miR-21 promotes cellular senescence by suppressing high-mobility group A2 in endothelial progenitor cells (EPCs) [16]. Conversely, miR-34a exerts controversial functions in cancer development and the aging process, as it functions as a tumor suppressor and a senescence inducer [17, 18]. miR-34a inhibits cancer progression by inhibiting cancer cell survival, proliferation, invasion, and metastasis formation [8, 9], while it promotes renal cell senescence by inhibiting antioxidant enzymes [19]. miR-34a induces cellular senescence through suppression of the longevity gene sirtuin 1 (SIRT1) in endothelial cells [18]. Overexpression of miR-34a induces premature senescence of young mesangial cells via downregulation of SOD2, leading to an increase in ROS generation, while antisense miR-34a inhibits senescence of old mesangial cells via upregulation of SOD2 and a decrease in ROS [19]. Collectively, these findings indicate that miR-21 and miR-34a are pivotal players in aging and tumor progression.

Kallistatin was first identified in human plasma as a tissue kallikrein inhibitor and a serine proteinase inhibitor [20–23]. Kallistatin is widely distributed in tissues relevant to cardiovascular function, including those of the kidney, heart, and blood vessels [24–26]. Kallistatin contains two structural elements: an active site and a heparin-binding domain [27–29]. The active site of kallistatin is a key for inhibiting tissue kallikrein activity and stimulating endothelial nitric oxide synthase (eNOS) and SIRT1 expression [30, 31]. Kallistatin via its heparin-binding site interacts with cell surface heparan sulfate proteoglycans, thus antagonizing signaling pathways mediated by vascular endothelial growth factor (VEGF), tumor necrosis factor- (TNF-) *α*, high-mobility group box-1 (HMGB1), TGF-*β*, and epidermal growth factor (EGF) [31–35]. Kallistatin administration exhibits pleiotropic effects, including reduction in blood pressure and inhibition of oxidative stress, inflammation, angiogenesis, apoptosis, hypertrophy, and fibrosis in animal models [33, 36–42]. Kallistatin inhibits TNF-*α*-induced ROS production, nuclear factor- (NF-) *κ*B activation, and inflammatory gene expression in endothelial cells [33, 42]. In addition, kallistatin reduces TNF-*α*-induced endothelial senescence and delays stress-induced aging by inhibiting miR-34a, miR-21, and oxidative stress and upregulating the expression of the antioxidant enzymes eNOS, SIRT1, catalase, and SOD3 in cultured EPCs, streptozotocin- (STZ-) induced diabetic mice, and *Caenorhabditis elegans* (*C. elegans ***)**. Moreover, kallistatin treatment prevents TGF-*β*-induced EndMT by reducing ROS formation and miR-21 synthesis [31]. Furthermore, kallistatin induces apoptosis by stimulating miR-34a and suppressing miR-21 synthesis in breast cancer cells [43]. This review focuses on the protective role of kallistatin in cellular senescence, aging, and tumor progression by inhibiting oxidative stress and regulating miR-34a and miR-21 levels.

## 2. Kallistatin Inhibits Oxidative Stress and Inflammation

Oxidative stress stimulates inflammatory pathways, which promotes cellular senescence and aging [44]. Besides superoxide radical (O_2_^−^), NF-*κ*B activation also links to accelerated aging in systemic inflammatory responses [45]. Oxidative stress and activation of NF-*κ*B have been shown to be associated with senescence of cultured endothelial cells, and pharmacological inhibition of NF-*κ*B signaling prevents age-associated features in mouse models [46]. Reduced kallistatin levels are correlated with increased oxidative stress, inflammation, and organ damage in animal models of hypertension, cardiovascular, and renal damage [22, 35, 40, 42]. Kallistatin treatment reduces ROS generation, inflammation, and organ damage associated with increased eNOS and NO levels in several animal models, including acute and chronic myocardial damage, salt-induced hypertension, and sepsis [34, 39, 40, 42]. On the other hand, depletion of endogenous kallistatin by neutralizing antibody injection further increases oxidative stress, inflammation, and fibrosis and augments cardiovascular and renal damage in salt-induced hypertensive rats [47]. These findings indicate that kallistatin has a protective role in cardiovascular and renal dysfunction by inhibition of oxidative stress and inflammation.

Kallistatin is an antioxidant as its heparin-binding site is essential for blocking TNF-*α*-induced NADPH oxidase activity and expression in endothelial cells [33, 42]. In addition, kallistatin's active site is a key for stimulating the expression of the antioxidant enzymes eNOS, SIRT1, and catalase in endothelial cells and EPCs [31, 48]. Kallistatin inhibits NAD(P)H oxidase activity and ROS formation, partly by NO formation in cardiomyocytes [39]. Kallistatin attenuates vascular inflammation by antagonizing TNF-*α*- and HMGB1-mediated NF-*κ*B activation and expression of proinflammatory genes in endothelial cells [33, 34]. Moreover, kallistatin stimulates eNOS expression by interacting with the transcription factor Kruppel-like factor 4 and increases eNOS activity and NO generation by activating the phosphoinositide 3-kinase- (PI3K-) Akt signaling pathway [41]. Kallistatin not only stimulates eNOS expression but also prevents TNF-*α*-mediated inhibition of eNOS synthesis in endothelial cells [31]. NO production can inhibit inflammatory gene expression by preventing activation of NF-*κ*B [49]. These findings indicate that kallistatin, through its antioxidative and anti-inflammatory effects, protects against multiorgan damage and accelerated aging.

## 3. Kallistatin Reduces Vascular Senescence and Aging by Downregulating miR-34a and miR-21 Synthesis

miR-34a and miR-21 have emerged as important regulators of cellular senescence and aging [50]. miR-34a functions as a main senescence promoter by inhibiting the expression of SIRT1, a conservative longevity gene, through a miR-34a-binding site within the 3′-UTR of SIRT1 [51]. Likewise, miR-21 is involved in accelerating cellular senescence in EPCs [16]. Moreover, oxidative stress induces vascular injury and endothelial senescence, with the inflammatory cytokine TNF-*α* being the main contributor to ROS production [52]. SIRT1 accounts for vascular homeostasis by activating many antioxidant enzymes, such as eNOS, catalase, and manganese superoxide dismutase (MnSOD), to diminish ROS [53]. Upregulation of antioxidant proteins, such as eNOS, SIRT1, catalase, and MnSOD, protects against oxidative stress-mediated insults [54, 55]. Therefore, prosenescent miR-34a and miR-21, as well as antioxidant enzymes, underlie the molecular basis for endothelial senescence and aging-associated diseases.

**Figure 1:**
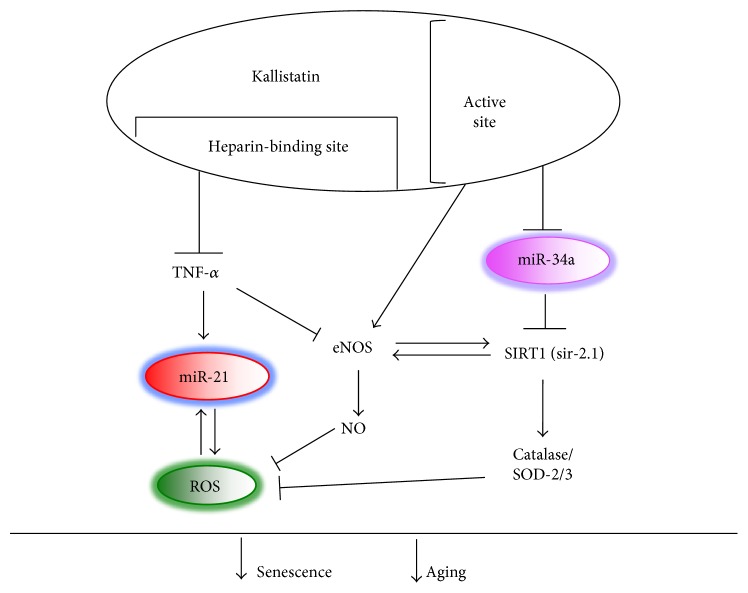
Signaling mechanism by which kallistatin inhibits senescence and aging through suppressing miR-34a expression and TNF-*α*-induced miR-21-ROS production and stimulating eNOS/SIRT1-NO levels in EPCs.

Kallistatin treatment significantly reduces TNF-*α*-induced senescence in EPCs, as indicated by reduced senescence-associated *β*-galactosidase activity and elevated telomerase activity. Telomerase prevents telomere attrition by synthesis of telomeric repeats onto the 3′ end of telomeres. Telomere shortening is observed during normal aging in humans and mice [56]. Circulatory kallistatin levels are positively associated with leucocyte telomere length in humans [57], indicating a potential role of kallistatin in maintaining telomere length and attenuating the aging process. In addition, kallistatin via its heparin-binding site antagonizes TNF-*α*-induced superoxide levels and miR-21 synthesis, as well as TNF-*α*-mediated inhibition of SIRT1, eNOS, and catalase synthesis in EPCs. Kallistatin via its active site inhibits miR-34a synthesis but stimulates the expression of antioxidant enzymes in EPCs. Overexpression of miR-34a abolishes kallistatin's effects on SIRT1 and eNOS and its antisenescence activity. Kallistatin administration attenuates aortic senescence, oxidative stress, and miR-34a and miR-21 synthesis, in association with elevated SIRT1, eNOS, and catalase levels in STZ-induced diabetic mice. Furthermore, human kallistatin treatment prolongs the lifespan of wild-type *C. elegans* under heat or oxidative stress conditions but has no effect on miR-34 or sir-2.1 (SIRT1 homolog) *C. elegans* mutants. Similar to diabetic mice, kallistatin inhibits miR-34 and superoxide formation but stimulates sir-2.1 synthesis in *C. elegans*. The signaling mechanism of kallistatin in senescence and aging by regulating miR-34a and miR-21 levels is shown in Figure 1. These combined findings provide significant insights into the novel role and mechanism of kallistatin in endothelial senescence and aging.

## 4. Kallistatin Inhibits EndMT via Suppressing TGF-*β*-Induced Oxidative Stress and miR-21 Expression

Endothelial-mesenchymal transition (EndMT) contributes greatly to organ fibrosis and tumor metastasis [58, 59]. Morphological changes in EndMT induced by TGF-*β* signaling cascades include loss of cell-cell junctions and endothelial markers, such as vascular endothelial- (VE-) cadherin and CD31, and gain of the mesenchymal marker *α*-smooth muscle actin (SMA). Moreover, miR-21 expression levels are highly elevated during EMT and EndMT [15, 60]. TGF-*β*-induced EndMT is partly mediated by upregulation of the miR-21-Akt pathway, as blockade of miR-21 can prevent EndMT and inhibit Akt activation [15]. Furthermore, ROS production further stimulates miR-21 synthesis in fibroblasts [61]. Therefore, miR-21 is a key mediator of EndMT, as well as EndMT-associated fibrosis and tumor development.

**Figure 2:**
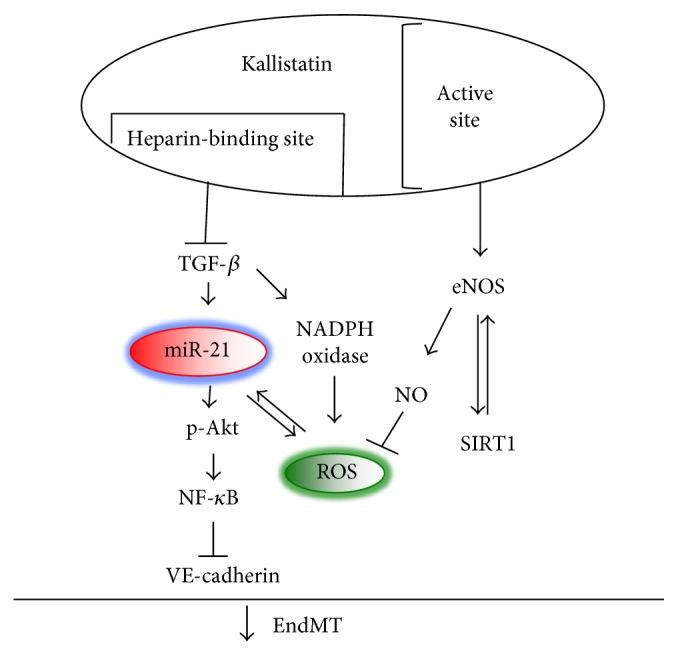
Signaling mechanism by which kallistatin inhibits EndMT by preventing the TGF-*β*-induced miR-21-Akt-NF-*κ*B pathway and oxidative stress and stimulating eNOS/SIRT1 expression in endothelial cells.

Human kallistatin delivery exerts beneficial effects on fibrosis by suppressing TGF-*β* synthesis in animal models [40, 62, 63]. Kallistatin treatment blocks TGF-*β*-induced EndMT in endothelial cells, as evidenced by morphological changes, increased endothelial markers (VE-cadherin and CD31), and reduced mesenchymal marker (*α*-SMA) [31]. Kallistatin prevents TGF-*β*-mediated activation of the miR-21-phospho-Akt-NF-*κ*B signaling pathway, as well as TGF-*β*-induced NADPH oxidase expression and activity, and ROS formation [31]. Kallistatin's heparin-binding site is crucial for inhibiting TGF-*β*-induced oxidative stress, while its active site is a key for stimulating expression of the antioxidant proteins eNOS and SIRT1 and production of NO. Moreover, kallistatin via the heparin-binding site blocks TGF-*β*-induced miR-21 expression, Akt phosphorylation, and NF-*κ*B activation. Thus, kallistatin inhibits EndMT through suppressing the TGF-*β*-induced miR-21-Akt-NF-*κ*B signaling pathway and stimulating antioxidant protein expression (Figure 2). These findings indicate that kallistatin attenuates fibrosis and cancer by suppressing TGF-*β*-induced EndMT.

## 5. Kallistatin Induces Cancer Cell Apoptosis by Stimulating miR-34a and Inhibiting miR-21 Expression

miRNAs are well categorized by their cancer-related functions, including tumor suppression, oncogene expression, epithelial-mesenchymal transition, apoptosis, and immune response [64]. miR-34a plays an important role as a tumor suppressor in many types of cancers [65]. Indeed, miR-34a levels are underexpressed in a variety of human tumors, and low levels of miR-34 have been related to poor clinical outcome of cancer patients [66, 67]. Moreover, miR-34a inhibits the proliferation, invasion, and migration of breast cancer cells and breast tumor growth in vivo by deactivating the Wnt/*β*-catenin signaling pathway [68]. On the other hand, miR-21, a well-recognized tumor inducer, is upregulated in a large range of human tumors, including gastric, colorectal, lung, pancreatic, ovarian, and breast cancer [69–74]. In addition, high levels of miR-21 expression are strongly related to poor clinical prognosis of patients in pancreatic cancer [75]. The prosurvival protein Bcl-2, a key regulator of apoptosis in many types of human tumors, is positively regulated by miR-21, and an anti-miR-21 inhibitor downregulates Bcl-2 in breast cancer cells [76]. Moreover, resveratrol induces bladder cancer cell apoptosis by reducing miR-21 expression, Akt phosphorylation, and Bcl-2 levels [77]. Therefore, these findings indicate opposite effects of miR-34a and miR-21 in cancer development.

**Figure 3:**
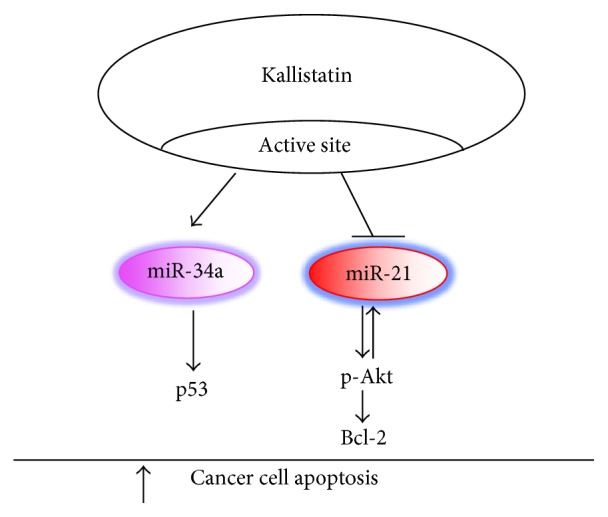
Signaling mechanism by which kallistatin induces apoptosis through upregulating miR-34a-p53 and downregulating miR-21-Akt-Bcl-2 pathways in breast cancer cells.

Kallistatin gene transfer has been reported to inhibit tumor growth and metastasis in several animal models [32, 78–82]. Local administration of human kallistatin reduces tumor growth and angiogenesis in nude mice via antagonizing VEGF-mediated proliferation, migration, and invasion of cultured endothelial cells [32, 78]. Moreover, kallistatin induces apoptotic cell death in human colorectal cancer cells [83]. Kallistatin via the active site stimulates miR-34a and suppresses miR-21 expression in breast cancer cells [44]. Kallistatin reduces cancer cell viability and induces apoptosis by increasing miR-34a and p53 expression but reducing miR-21 synthesis, Akt phosphorylation, and Bcl-2 expression in breast cancer cells [44]. Thus, kallistatin induces breast cancer cell apoptosis by stimulating miR-34a-p53 and suppressing miR-21-Akt-Bcl-2 signaling pathways (Figure 3). These findings indicate that kallistatin induces cancer cell death through upregulation of miR-34a and downregulation of miR-21 expression.

## 6. Conclusion

Kallistatin plays a protective role in accelerated aging and cancer development by regulation of miR-34a and miR-21. Kallistatin's antisenescence/aging effect is mainly attributed to suppression of oxidative stress and inflammation and downregulation of miR-34a and miR-21 synthesis. Kallistatin via its heparin-binding domain antagonizes TNF-*α*-induced ROS formation and miR-21 expression, while its active site is crucial for inhibiting miR-34a synthesis in EPCs. Kallistatin protects against fibrosis and tumorigenesis by inhibiting EndMT. Kallistatin's heparin-binding site is critical for suppressing TGF-*β*-induced ROS formation and the miR-21-Akt-NF-*κ*B signaling pathway in endothelial cells. However, kallistatin via its active site stimulates miR-34a and p53 synthesis and inhibits miR-21-Akt-Bcl-2 signaling, leading to apoptosis in breast cancer cells. As an endogenous protein, kallistatin treatment could improve human health during aging process and tumor progression with minimal side effects. This review article provides a new potential prospective in kallistatin-based therapeutic intervention in aging and cancer.
